# Analysis of Polynomial Nonlinearity Based on Measures of Nonlinearity Algorithms

**DOI:** 10.3390/s20123426

**Published:** 2020-06-17

**Authors:** Mahendra Mallick, Xiaoqing Tian

**Affiliations:** 1 Independent Consultant, Anacortes, WA 98221, USA; 2 School of Automation Science and Engineering, Xi’an Jiaotong University, Xi’an 710049, China; tianxiaoqing2017@stu.xjtu.edu.cn

**Keywords:** polynomial curve in 2D, measures of nonlinearity (MoNs), extrinsic curvature, Beale’s MoN, Linssen’s MoN, Bates and Watts parameter-effects curvature, direct parameter-effects curvature, Li’s MoN, MoN of Straka, Duník, and S̆imandl, maximum likelihood estimator (MLE), Cramér-Rao lower bound (CRLB)

## Abstract

We consider measures of nonlinearity (MoNs) of a polynomial curve in two-dimensions (2D), as previously studied in our Fusion 2010 and 2019 ICCAIS papers. Our previous work calculated curvature measures of nonlinearity (MoNs) using (i) extrinsic curvature, (ii) Bates and Watts parameter-effects curvature, and (iii) direct parameter-effects curvature. In this paper, we have introduced the computation and analysis of a number of new MoNs, including Beale’s MoN, Linssen’s MoN, Li’s MoN, and the MoN of Straka, Duník, and S̆imandl. Our results show that all of the MoNs studied follow the same type of variation as a function of the independent variable and the power of the polynomial. Secondly, theoretical analysis and numerical results show that the logarithm of the mean square error (MSE) is an affine function of the logarithm of the MoN for each type of MoN. This implies that, when the MoN increases, the MSE increases. We have presented an up-to-date review of various MoNs in the context of non-linear parameter estimation and non-linear filtering. The MoNs studied here can be used to compute MoN in non-linear filtering problems.

## 1. Introduction

The Kalman filter (KF) [1,2,3,4] is an optimal estimator (in the minimum mean square error (MMSE) sense) for a filtering problem with linear dynamic and measurement models with additive Gaussian noise. However, many real-world filtering problems are non-linear due to nonlinearity in the dynamic and measurement models. Common real-world non-linear filtering (NLF) problems are bearing-only filtering [5,6,7,8], ground moving target indicator (GMTI) filtering [9], passive angle-only filtering in three-dimensions (3D) using an infrared search and track sensor [10,11,12], etc.

In the early stages of NLF, the extended Kalman filter (EKF) [1,2,3,4] was widely used. It was observed in some problems, e.g., falling of a body in earth’s atmosphere with high velocity [13,14] and bearing-only filtering [5,7,8] that the EKF performs poorly due to linearization. The high degree of nonlinearity in these problems was the attributed cause for the poor performance of the problem without a quantitative measure of nonlinearity (MoN). To overcome the poor accuracy and convergence problems of the EKF, a number of improved approximate non-linear filters, such as the unscented Kalman filter (UKF) [14,15], cubature KF (CKF) [16], and particle filter (PF) [8,17] have been proposed during the last two decades.

It is important to address the following questions for NLF problems:

1. Is it possible to find a quantitative MoN for a nonlinear filtering problem?
2. Can we establish a correspondence between the MoN of a NLF problem and the performance of a filtering algorithm?
3. Can we show that the UKF, CKF, or PF gives better results than the EKF, when the degree of nonlinearity (DoN) is high?

**Remark** **1.** *In this paper we consider a parameter estimation problem with polynomial nonlinearity. We hope that insights and results from this analysis would encourage further study of MoN in NLF problems. Next, we describe some historical developments in the field of parameter estimation and NLF.*

Beale in his pioneering work [18] proposed four MoNs for the static non-random parameter estimation problem. Two MoNs were empirical and two were theoretical. Guttman and Meeter [19] and Linssen [20] observed that Beale’s method gives lower MoN for highly non-linear problems and proposed a modified MoN. Using differential geometry based curvature measures, Bates and Watts [21,22] and Goldberg et al. [23] extended Beale’s work and developed curvature measures of nonlinearity (CMoN) for the static non-random parameter estimation problem. Bates and Watts formulated two CMoN, the parameter-effects curvature and intrinsic curvature [21,24,25,26].

In [27], we first extended the method of Bates and Watts to the non-linear filtering problem with unattended ground sensor (UGS) to calculate CMoN. Next, we computed the parameter-effects curvature and intrinsic curvature for the bearing-only filtering (BOF) problem [28,29,30,31], GMTI filtering problem [30,32,33], video tracking problem [34], and polynomial nonlinearity [35].

In our previous work [35], we considered a polynomial curve in two-dimensions (2D) and calculated CMoN using differential geometry (e.g., extrinsic curvature) [36,37,38], Bates and Watts parameter-effects curvature [21,25,26], and direct parameter-effects curvature [29]. The computation of these curvatures requires the Jacobian and Hessian of the measurement function [2] evaluated at the true or estimated parameter. The extrinsic curvature uses the true parameter, whereas the other two CMoN use the estimated parameter.

In [35], we obtained the maximum likelihood (ML) estimate [2,39] of the parameter *x* while using a vector measurement by numerical minimization. In [40], we derived analytic expressions for the ML estimator (MLE) [2,39] and associated variance using a vector measurement. This approach is simple and efficient, since it does not require numerical minimization. We also showed through Monte Carlo simulations in [40] that the variance of the MLE and the Cramér-Rao lower bound (CRLB) [2,41] are nearly the same for different powers of *x*. We also found that the bias error was small and the mean square error (MSE) [2] was close to the CRLB and variance of the MLE. Our numerical results showed that the average normalized estimation error squared (ANEES) [42] was within the 99% confidence interval most of the time. Hence, the variance of the MLE was in agreement with the estimation error.

Li constructed a combined non-linear function while using the non-linear time evolution function and measurement function in a discrete-time nonlinear filtering problem, and he proposed a global MoN at each measurement time [43]. This MoN minimizes the mean square distance between the combined non-linear function and the set of all affine functions with the same dimension at each measurement time. An un-normalized MoN and a normalized MoN were proposed in [43]. These MoNs can also be unconditional or conditional. The normalized MoN lies in the interval [0,1]. A journal version of the paper with enhancements was published in [44].

The normalized MoN that was proposed in [43] was calculated for non-linear filtering problems, including one with the nearly constant turn motion and a non-linear measurement model [45], a video tracking problem using PF [46], and a hypersonic entry vehicle state estimation problem [47]. In these cases, the normalized MoN were rather low. In [33], we compared the normalized MoN for the BOF and GMTI filtering problems. Contrary to our expectation, we found that the GMTI filtering problem had a higher conditional normalized MoN than that of the BOF problem in the examples that we investigated.

Using the current mean (e.g., predicted mean) and associated covariance, Duník et al. [48] generate a number of sample points (e.g., sigma points using unscented transform [14]) and transform these points using a non-linear function (e.g., non-linear measurement function or time evolution function). Subsequently, they try to predict the transformed points using a linear transformation and estimate the parameter of the transformation using linear weighted least squares (WLS) [39]. They use the cost function of the WLS evaluated at the estimated parameter as a local MoN.

In [35], we showed analytically and through Monte Carlo simulations that affine mappings with positive slopes exist among the logarithm of the extrinsic curvature, Bates and Watts parameter-effects curvature, direct parameter-effects curvature, MSE, and CRLB. For completeness, we have included these key results from [35] in Section 4. New contributions in this paper include the computation and analysis of following MoNs:

- Beale’s MoN [18],
- Least squares based Beale’s MoN,
- Linssen’s MoN [20],
- Least squares based Linssen’s MoN,
- Li’s MoN [43,44], and
- MoN of Straka, Duník, and S̆imandl [48,49].

It is not possible to derive a mapping analytically between the logarithm of Beale’s MoN, Linssen’s MoN, Li’s MoN, MoN of Straka, Duník, and S̆imandl, and the logarithm of the MSE. The numerical results from Monte Carlo simulations also show that affine mappings with positive slopes exist among the logarithm of the MSE and the logarithm of two of these MoNs.

The paper is organized, as follows. Section 2.1 describes the measurement model for polynomial nonlinearity. The MLE for parameter estimation and CRLB using polynomial nonlinearity and a vector measurement is presented in Section 2. Section 3 presents different types of MoN, such as extrinsic curvature based on differential geometry, Beale’s MoN, Linssen’s MoN, Bates and Watts parameter-effects curvature, direct parameter-effects curvature, Li’s MoN, and MoN of Straka, et al. Section 4 discusses mappings among logarithms of extrinsic curvature, parameter-effects curvature, CRLB, and MSE. Section 5 presents the numerical simulation and results. Finally, Section 6 summarizes our contribution and concludes with future work.

*Notation Convention:* For clarity, we use italics to denote scalar quantities and boldface for vectors and matrices. A lower or upper case Roman letter represents a name (e.g., “s” for “sensor”, “RMS” for “root mean square”, etc.). We use “:=” to define a quantity and $A^{\prime }$ denotes the transpose of the vector or matrix A. The n−dimensional identity matrix, m−dimensional null matrix, and m×n null matrix are denoted by $I_{n}$, $0_{m}$, and $0_{m\times n}$, respectively.

## 2. MLE Parameter Estimation and CRLB

### 2.1. Measurement Model

We studied CMoN of a polynomial smooth scalar function *h* of a non-random variable *x* in [35], where

(1) $$h\left(x\right)=ax^{n},$$

and *a* is a non-zero scalar. In scenarios considered, x>0 and n=2,3,4,5.

**Remark** **2.** *For MoN of other forms of nonlinearity, such as the bearing-only [27], GMTI [32], and video filtering [34] problems in radar communities, we shall discuss in detail in the end of Section 3.*

The measurement model for the polynomial function is given by

(2) $$z_{i}=h\left(x\right)+v_{i},\;i=1,\ldots ,N,$$

where $v_{i}$ is a zero-mean white Gaussian measurement noise with variance $\sigma^{2}$,

(3) $$v_{i}∼N\left(0,\sigma^{2}\right).$$

We assume that the measurement noises are independent.

The measurement model can be written in the vector form

(4) z=h(x)+v,

where

(5) $$z:={\left[\begin{array}{cccc} z_{1} & z_{2} & \ldots & z_{N} \end{array}\right]}^{\prime },$$

(6) $$v:={\left[\begin{array}{cccc} v_{1} & v_{2} & \ldots & v_{N} \end{array}\right]}^{\prime },$$

(7) h(x):=h(x)d,

(8) $$d:={\left[\begin{array}{cccc} 1 & 1 & \ldots & 1 \end{array}\right]}^{\prime },$$

(9) $$v∼N\left(0,R\right),\;R=I_{N}\sigma^{2}.$$

### 2.2. ML Estimate of Parameter

The likelihood function of *x* is [2,50,51]

(10) $$\begin{array}{c} {\Lambda \left(x;z\right)=p\left(z|x\right)=[\left(2\pi \right)}^{N}{\left|R|\right]}^{-1/2}\exp\left\{-\left(1/2\right){\left[z-h\left(x\right)\right]}^{\prime }R^{-1}\left[z-h\left(x\right)\right]\right\}. \end{array}$$

The maximization of the likelihood in (10) is equivalent to the minimization of the cost function [2,51]

(11) $$\begin{array}{c} J\left(x\right)={\left[z-h\left(x\right)\right]}^{\prime }R^{-1}\left[z-h\left(x\right)\right]={\left[z-h\left(x\right)\right]}^{\prime }\left[z-h\left(x\right)\right]/\sigma^{2}. \end{array}$$

The maximum likelihood (ML) estimate $\hat{x}$ of *x* is obtained by setting the derivative of J(x) to zero [2,51],

(12) $$\frac{dJ(x)}{dx}=0.$$

From (11) and (12), we obtain

(13) $${\left[z-h\left(\hat{x}\right)\right]}^{\prime }\frac{dh(\hat{x})}{dx}=0.$$

Because the derivative of h(x) with respect to *x* is not zero, we obtain

(14) $$z-h\left(\hat{x}\right)=0_{N\times 1}.$$

Hence, the ML estimate satisfies,

(15) $$h(\hat{x})d=z.$$

Left-multiplying both sides of (15) by $d^{\prime },$ we obtain

(16) $$h\left(\hat{x}\right)d^{\prime }d=d^{\prime }z=\sum_{i=1}^{N}z_{i}.$$

We note that

(17) $$d^{\prime }d=N.$$

Using (1) and (17) in (16) we get

(18) $$a{\hat{x}}^{n}=\bar{z},$$

where $\bar{z}$ is the sample mean of *z*,

(19) $$\bar{z}=\frac{1}{N}\sum_{i=1}^{N}z_{i}.$$

Thus, from (18), the ML estimate of *x* is given by

(20) $$\hat{x}={\left(\bar{z}/a\right)}^{1/n},\;n=2,3,\ldots .$$

**Remark** **3.** *In general, the MLE for a nonlinear measurement model is biased [51]. We can calculate the variance of $\hat{x}$ under the small error assumption using the linearization approximation. To guarantee the validity of the variance, the bias in the MLE must be calculated. The bias can be numerically calculated using Monte Carlo simulation.*

*The bias in the MLE is defined by [2,51]*

(21) $$b\left(x\right):=x-\hat{x}.$$

**Remark** **4.** *The ML estimate of x in [35] was obtained by minimizing the cost function in (11) numerically. The estimator in (20) provides simple and efficient way of estimating x using a vector measurement z without numerical optimization.*

### 2.3. Variance of the MLE

The variance of $\hat{x}$ is given by [51]

(22) $$\sigma_{x}^{2}={\left({\dot{H}}^{\prime }R^{-1}\dot{H}\right)}^{-1},$$

where

(23) $$\dot{H}=\frac{dh(x)}{dx}{|}_{x=\hat{x}}.$$

Using the special form of R from (9) in (22), we get

(24) $$\sigma_{x}^{2}=\sigma^{2}{\left({\dot{H}}^{\prime }\dot{H}\right)}^{-1}.$$

Using (7) in (23), we get

(25) $$\dot{H}=\frac{dh(x)}{dx}{|}_{x=\hat{x}}\;d.$$

Differentiating (1) with respect to *x*, we obtain

(26) $$\frac{dh(x)}{dx}=anx^{n-1}.$$

Using (26) in (25), we get

(27) $$\dot{H}=an{\hat{x}}^{n-1}d.$$

From (27), we obtain

(28) $${\dot{H}}^{\prime }\dot{H}={\left(an{\hat{x}}^{n-1}\right)}^{2}\;d^{\prime }d,$$

Using (28) and (17) in (24), we obtain

(29) $$\sigma_{x}^{2}=\sigma^{2}{\left({\dot{H}}^{\prime }\dot{H}\right)}^{-1}=\frac{\sigma^{2}}{N{\left(an{\hat{x}}^{n-1}\right)}^{2}},$$

(30) $$\sigma_{x}=\frac{\sigma }{\sqrt{N}an{\hat{x}}^{n-1}}.$$

### 2.4. Cramér-Rao Lower Bound

The CRLB [2,41] for the MSE in the current problem is given by

(31) $${\mathrm{CRLB}}_{x}={\left[\frac{dh^{\prime }\left(x\right)}{dx}R^{-1}\frac{dh(x)}{dx}\right]}^{-1}.$$

**Remark** **5.** *Calculation of the variance $\sigma_{x}^{2}$ and ${\mathrm{CRLB}}_{x}$ are similar. For $\sigma_{x}^{2}$, we use the estimate $\hat{x}$ while calculating the Jacobian of the measurement function, whereas, for ${\mathrm{CRLB}}_{x}$, we use the true x while calculating the Jacobian of the measurement function.*

Using similar procedure, we obtain

(32) $${\mathrm{CRLB}}_{x}=\frac{\sigma^{2}}{N{\left(anx^{n-1}\right)}^{2}},$$

(33) $$\sqrt{{\mathrm{CRLB}}_{x}}=\frac{\sigma }{\sqrt{N}anx^{n-1}}.$$

From (30) and (33), we find that, for a given *x*, the standard deviation (SD) and square root of CRLB are inversely proportional to the power *n*. Secondly (33) shows that, for a given power, the square root of CRLB decreases as *x* increases.

## 3. Measures of Nonlinearity

**Figure 1:**
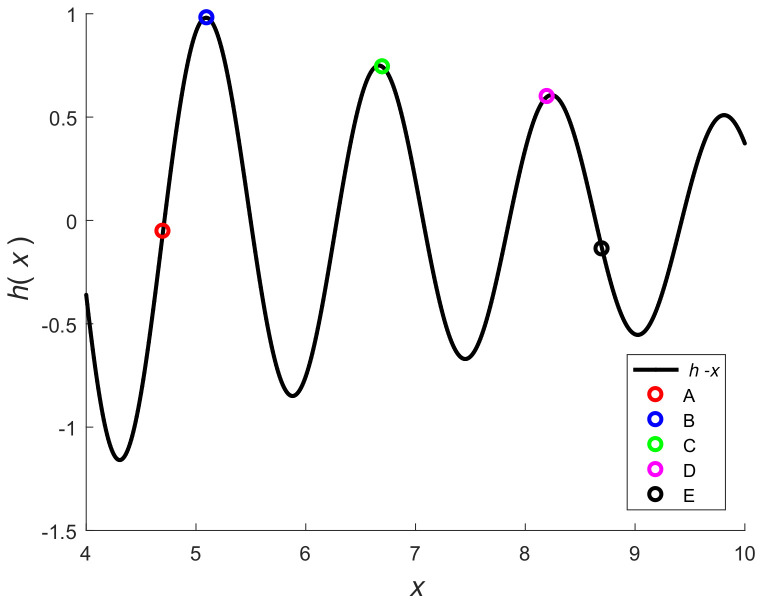
y=5sin(4x)/x versus *x*.

To explain the key concepts of nonlinearity, consider the scalar function h(x)=5sin(4x)/x shown in Figure 1. We observe in Figure 1 that the function is nearly linear at A and E. If we draw a tangent to the curve at A and E, then the curve is close to the tangent in the neighborhood of A and E. However, tangents to the curve at points B, C, and D differ by large amounts from the curve in the neighborhood of these points. The tangent represents an affine approximation to the curve at a point. We observe that, among points B, C, and D, the curve bends the most at B and the least at point D. If we draw a circle (called the osculating circle) at these points, then the radius of the circle can be used to judge nonlinearity. The rate of bending is high when the radius of the circle is small. In differential geometry [37,38], the curvature κ is inverse of the radius of the osculating circle and, hence, curvature can be viewed as a measure of nonlinearity. The radii of the osculating circles at A and E are nearly infinity and, hence, the curvatures are nearly zero. From Figure 1, we observe that, in general, the nonlinearity of a function can vary with *x*. Hence, the nonlinearity is a local measure. If the second derivative of a function is non-zero, then the function is non-linear.

In [35,40], we analyzed the CMoN of a polynomial scalar function *h* of a non-random variable *x*, as described in Section 2.1. The CMoN were based on the extrinsic curvature using differential geometry, Bates and Watts parameter-effects curvature, and direct parameter-effects curvature. In this paper, we study the following MoNs:

- extrinsic curvature using differential geometry [36,37,38],
- Beale’s MoN [18],
- least squares based Beale’s MoN,
- Linssen’s MoN [20],
- Least squares based Linssen’s MoN,
- parameter-effects curvatures [21,25,29],
- Li’s MoN [43,44], and
- MoN of Straka, Duník, and S̆imandl [48,49].

If a MoN has a high value, then the nonlinearity is high and if it has a low value, then the Therefore, it is impossible to compare them based on numerical values. We can only study their variations.

Consider the *m*-dimensional vector non-linear function h of the non-random n−dimensional parameter x. Let $\hat{x}$ be a known estimate of x. Using the Taylor series expansion of h(x) about $\hat{x}$ and keeping the first order term gives

(34) $$h\left(x\right)\approx T\left(x\right)=h\left(\hat{x}\right)+\dot{H}\left(x-\hat{x}\right),$$

where T(x) represents the tangent plane approximation (an affine mapping) to h(x) and

(35) $$\dot{H}=\frac{dh(x)}{dx}{|}_{x=\hat{x}}.$$

If m>n, then h is an n−dimensional manifold embedded in an m−dimensional space [37,38]. The tangent plane is tangent to the surface h at $\hat{x}$. The concept of tangent plane is used in Beale’s MoN, Linssen’s MoN, Bates and Watts parameter-effects curvatures [21,25], and direct parameter-effects curvature [44].

For polynomial nonlinearity, the CMoN using differential geometry is calculated at the true value *x* and, hence, it is non-random. The Bates and Watts parameter-effects curvature, direct parameter-effects curvature, Beale’s MoN, Li’s MoN, and the MoN of Straka et al. are calculated while using an estimate $\hat{x}$ of *x*. The estimate $\hat{x}$ is obtained from a measurement model involving the measurement function *h*. Since *x* is a scalar, we need one or more scalar measurements to estimate *x*. Table 1 summarizes features of various MoNs.

The CMoN using differential geometry [36,37,38] is calculated at the true value *x*, whereas the Bates and Watts parameter-effects curvature [21,25,26], direct parameter-effects curvature [29], Beale’s MoN, Li’s MoN, and the MoN of Straka et al. are calculated while using an estimate $\hat{x}$ of *x*. The estimate $\hat{x}$ is obtained from a measurement model involving the measurement function *h*. Since *x* is a scalar, we need one or more scalar measurements to estimate *x*. Next, we describe various MoN.

### 3.1. Extrinsic Curvature Using Differential Geometry

The curvature of a circle at every point on the circumference is equal to the inverse of the radius of the circle. Thus, the curvature of a circle is a constant. A circle with a smaller radius bends more sharply and, therefore, has a higher curvature.

We assume that the first and second derivatives of the nonlinear smooth scalar function *h* exist. The curvature of the curve y=h(x) at a point *x* is equal to the curvature of the osculating circle at that point. The extrinsic curvature at the point *x* is defined by [36,37,38],

(36) $$\kappa \left(x\right):=\frac{\left|\frac{d^{2}h\left(x\right)}{dx^{2}}\right|}{{\left[1+{\left(\frac{dh(x)}{dx}\right)}^{2}\right]}^{3/2}}=\frac{\left|\ddot{h}\left(x\right)\right|}{{\left[1+\dot{h}{\left(x\right)}^{2}\right]}^{3/2}}.$$

The first derivative of *h* at a point *x* is given in (26). The second derivative of *h* with respect to *x* is given by

(37) $$\ddot{h}\left(x\right)=\frac{d^{2}h\left(x\right)}{dx^{2}}=an\left(n-1\right)x^{n-2},\;n=2,3,\ldots .$$

Thus, using $\dot{h}\left(x\right)$ and $\ddot{h}\left(x\right)$ in (36), we can calculate the extrinsic curvature κ(x) at any point *x* by

(38) $$\kappa \left(x\right)=\frac{an\left(n-1\right)x^{n-2}}{{\left[1+{\left(an\right)}^{2}x^{2(n-1)}\right]}^{3/2}}.$$

### 3.2. Beale’s MoN

Consider the nonlinear measurement model for the non-random *n*-dimensional parameter x

(39) z=h(x)+v,

where z, h, and v are the measurement, non-linear measurement function, and measurement noise, respectively. Let $\hat{x}$ be an estimate of x. Subsequently, a Taylor series expansion of h(x) about $\hat{x}$ and keeping the first order term is as (34). Suppose we choose *m* vectors $x_{i},i=1,2,\ldots ,m$ in the neighborhood of x. Then Beale’s first empirical MoN [18] is given by

(40) $${\hat{N}}_{x}=\rho^{2}\frac{\sum_{i=1}^{m}{∥h\left(x_{i}\right)-T\left(x_{i}\right)∥}^{2}}{\sum_{i=1}^{m}{∥h\left(x_{i}\right)-h\left(\hat{x}\right)∥}^{4}},$$

where ρ is the standard radius and it is defined by

(41) $$\rho^{2}:={∥z-h\left(\hat{x}\right)∥}^{2}/\left(n\left(N-n\right)\right).$$

Guttman and Meeter [19] observed that the empirical MoN underestimates severe nonlinearity. When *m* approaches infinity, the empirical MoN ${\hat{N}}_{x}$ approaches the theoretical MoN $N_{x}.$

### 3.3. Least Squares Based Beale’s MoN

Consider the scalar function *h* for polynomial nonlinearity, as described in (1). As described in Beale’s MoN, we choose *m* points $x_{i},i=1,2,\ldots ,m$ in th neighborhood of x. Let

(42) $$y_{i}=ax_{i}^{n},\;i=1,2,\ldots ,m.$$

An affine mapping as approximation to $y_{i}$ is given by

(43) $$L\left(x_{i}\right)=A+Bx_{i},\;i=1,2,\ldots ,m.$$

We compute *A* and *B* by minimizing the cost function

(44) $$J\left(A,B\right):=\sum_{i=1}^{m}{\left(y_{i}-A-Bx_{i}\right)}^{2}$$

by the method of least squares (LS) [2,39]. The LS minimization of the cost function yields [52]

(45) $$\hat{B}=\left(C_{xy}-\bar{x}\;\bar{y}\right)/\left(C_{xx}-{\bar{x}}^{2}\right),$$

(46) $$\hat{A}=\bar{y}-\hat{B}\bar{x},$$

where

(47) $$\bar{x}=\frac{1}{m}\sum_{i=1}^{m}x_{i},\;\bar{y}=\frac{1}{m}\sum_{i=1}^{m}y_{i},$$

(48) $$C_{xx}=\frac{1}{m}\sum_{i=1}^{m}x_{i}^{2},\;C_{xy}=\frac{1}{m}\sum_{i=1}^{m}x_{i}y_{i}.$$

Then we can use the affine mapping with $\hat{A}$ and $\hat{B}$ in Beale’s MoN.

### 3.4. Linssen’s MoN

In order the correct the deficiency in Beale’s MoN, Linssen proposed a modification to obtain the following MoN [20]

(49) $$M^{*}=\sqrt{\rho^{2}\frac{\sum_{i=1}^{m}{∥h\left(x_{i}\right)-T\left(x_{i}\right)∥}^{2}}{\sum_{i=1}^{m}{∥h\left(\hat{x}\right)-T\left(x_{i}\right)∥}^{4}}}.$$

### 3.5. Least Squares Based Linssen’s MoN

Using the same procedure as in Section 3.3, we can use an affine mapping with $\hat{A}$ and $\hat{B}$ as an approximation to $y_{i}$ in computing Linssen’s MoN.

### 3.6. Parameter-Effects Curvatures

The parameter-effects curvature and intrinsic curvature defined by Bates and Watts [21,25,26] are associated with a non-linear parameter estimation problem and are defined at the estimated parameter. We note that in (1), h:R→R. Since *h* is a scalar function, the intrinsic curvature of Bates and Watts $K^{N}\left(\hat{x}\right)$ [21] or the direct intrinsic curvature $\beta_{\delta }^{N}\left(\hat{x}\right)$ [29] is zero. Thus, only the parameter-effects curvature of Bates and Watts $K^{T}\left(\hat{x}\right)$ and the direct parameter-effects curvature $\beta_{\delta }^{T}\left(\hat{x}\right)$ are non-zero. Since the intrinsic curvature is zero, for simplicity in notation, we drop the superscript “T” from the parameter-effects curvature and they are given by

(50) $$K\left(\hat{x}\right):=\frac{\left|\right|\ddot{H}\delta^{2}\left|\right|}{\left|\right|\dot{H}\delta {\left|\right|}^{2}}=\frac{\left|\right|\ddot{H}\left|\right|}{\left|\right|\dot{H}{\left|\right|}^{2}},$$

(51) $$\beta_{\delta }\left(\hat{x}\right):=\frac{\left|\right|\ddot{H}\delta^{2}\left|\right|}{\left|\right|\dot{H}\delta \left|\right|}=\frac{|\ddot{H}\left||\delta \right|}{\left|\right|\dot{H}\left|\right|},$$

where

(52) $$\ddot{H}=\frac{d^{2}h\left(x\right)}{dx^{2}}{|}_{x=\hat{x}},$$

(53) $$\delta :=x-\hat{x}.$$

From (26), we get

(54) $$\ddot{H}=an\left(n-1\right){\hat{x}}^{n-2}d.$$

Hence, from (27) and (52), we obtain

(55) $$\left|\right|\dot{H}\left|\right|=an{\hat{x}}^{n-1}\;\sqrt{N}.$$

(56) $$\left|\right|\ddot{H}\left|\right|=an\left(n-1\right){\hat{x}}^{n-2}\sqrt{N}.$$

Substitution of results from (55) and (56) in (50) and (51) gives

(57) $$K\left(\hat{x}\right)=\frac{n-1}{na\sqrt{N}}\;\frac{1}{{\hat{x}}^{n}},$$

(58) $$\beta_{\delta }\left(\hat{x}\right)=\frac{\left(n-1)|\delta \right|}{\hat{x}}.$$

We note that the extrinsic curvature in (36) is evaluated at the true *x*, while the parameter-effects curvatures $K(\hat{x})$ in (50) and $\beta_{\delta }\left(\hat{x}\right)$ in (51) are evaluated at the estimate $\hat{x}$. Because $\hat{x}$ is a random variable, $K(\hat{x})$ and $\beta_{\delta }\left(\hat{x}\right)$ are random variables. When we perform Monte Carlo simulations and estimate *x* from measurements, $\hat{x}$ varies among Monte Carlo runs. Therefore, $K(\hat{x})$ and the set of all linear $\beta_{\delta }\left(\hat{x}\right)$ vary with Monte Carlo runs.

### 3.7. Li’s MoN

For a scalar random variable *x*, the un-normalized MoN proposed by Li [43,44] represents the square root of the minimum mean square distance between the nonlinear measurement function *h* and the set of all affine functions *L*,

(59) $$J=\min{\left(E\left\{{\left(L\left(x\right)-h\left(x\right)\right)}^{2}\right\}\right)}^{1/2},$$

where L(x)=Ax+B. The scalar parameters *A* and *B* are determined in the minimization process. For the current problem, where *x* is non-random, the un-normalized MoN *J* and normalized MoN ν ar given, respectively, by

(60) $$J=\sigma_{h}\sqrt{1-\frac{c_{hx}^{2}}{\sigma_{h}^{2}\sigma_{x}^{2}}}.$$

(61) $$\nu =J/\sigma_{h}=\sqrt{1-\frac{c_{hx}^{2}}{\sigma_{h}^{2}\sigma_{x}^{2}}}.$$

Given $\hat{x}$ and $\sigma_{x}$ (30), the unscented transformation (UT) [14,15], cubature transformation (CT) [16], or Monte Carlo method [8] can be used to compute $\sigma_{h}^{2}$ and $c_{hx}$. We find that the UT gives good results in calculating the two MoNs. Next we dscribe computing *J* and ν using the UT. We use $\kappa_{\mathrm{UT}}=2$ [14]. The three weights and sigma points are given, respectively, by

(62) $$w_{0}=2/3,\;w_{1}=1/6,\;w_{2}=1/6,$$

(63) $$\chi_{0}=\hat{x},\;\chi_{1}=\hat{x}+\sqrt{3}\sigma_{x},\;\chi_{2}=\hat{x}-\sqrt{3}\sigma_{x}.$$

The measurement transformed points are

(64) $$h_{i}=a\;\chi_{i}^{n},\;i=0,1,2.$$

Then the mean and variance of *h* a given by

(65) $$\bar{h}=\sum_{i=0}^{2}w_{i}h_{i},$$

(66) $$\sigma_{h}^{2}=\sum_{i=0}^{2}w_{i}{\left(h_{i}-\bar{h}\right)}^{2},$$

The cross-covariance $c_{hx}$ is computed by

(67) $$c_{hx}=\sum_{i=0}^{2}w_{i}\left(h_{i}-\bar{h}\right)\left(\chi_{i}-\hat{x}\right).$$

### 3.8. MoN of Straka, Duník, and  S̆imandl

Straka, Duník, and S̆imandl presented two local MoNs in [48,49]. Given the estimate $\hat{x}$ and variance $\sigma_{x}^{2},$ these MoNs use a number of points $\chi_{i},i=1,2,\ldots ,m$ in the neighborhood of $\hat{x}.$ We analyze the first MoN proposed by the authors. The transformed points by the non-linear function *h* are given by

(68) $$z_{i}=h\left(\chi_{i}\right),\;i=1,2,\ldots ,m.$$

Define

(69) $$Z:={\left[\begin{array}{cccc} z_{1} & z_{2} & \cdots & z_{m} \end{array}\right]}^{\prime },$$

(70) $$X:={\left[\begin{array}{cccc} \chi_{1} & \chi_{2} & \cdots & \chi_{m} \end{array}\right]}^{\prime }.$$

A linear approximation to Z is Xθ, where θ is a scalar parameter to be estimated. The cost function that is proposed in [48,49] to determine θ is given by

(71) $$J_{1}\left(\theta \right):={\left(Z-X\theta \right)}^{\prime }W\left(Z-X\theta \right),$$

where the weight-matrix W is given by

(72) $$W=\mathrm{diag}(d_{1},d_{2},\ldots ,d_{m}),$$

(73) $$d_{i}={\left(\chi_{i}-\hat{x}\right)}^{2},\;i=1,2,\ldots ,m.$$

The LS estimate [39] that minimizes the cost function is given by

(74) $${\hat{\theta }}_{\mathrm{LS}}={\left(X^{\prime }WX\right)}^{-1}X^{\prime }WZ.$$

For this problem, the LS estimate in (74) reduces to

(75) $${\hat{\theta }}_{\mathrm{LS}}={\left(\sum_{i=1}^{m}d_{i}\chi_{i}^{2}\right)}^{-1}\sum_{i=1}^{m}d_{i}\chi_{i}z_{i}.$$

The cost function $J_{1}$ evaluated at ${\hat{\theta }}_{\mathrm{LS}}$ is treated as a local MoN η, given by

(76) $$\eta =J_{1}\left({\hat{\theta }}_{\mathrm{LS}}\right).$$

**Remark** **6.** *We have calculated the average MoN for the bearing-only filtering [27], GMTI [32], and video filtering [34] problems. The MoN is presented in the table below (Table 2). From this table we find that the degree of nonlinearity of the bearing-only filtering problem is about two orders of magnitude higher than that of the GMTI or video filtering problem. This implies that a simple filter, such as the EKF or UKF, is sufficient for the GMTI or video filtering problem, but an advanced filter, such as the PF, is needed for the BOF [17] problem.*

## 4. Mapping between CMoN and MSE in Polynomial NonLinearity

The nonlinearity of the problem imposes challenges in parameter estimation. We analyze the CMoN and MSE of the non-linear estimation problem to discover relationships among them. For the current problem, CMoN are measured by the parameter-effects curvature in (57) and the direct parameter-effects curvature in (58). In general, CMoN depend on the first and second derivatives of the non-linear function calculated at the parameter estimate and on the norm of the estimation error for $\beta_{\delta }\left(\hat{x}\right)$. Therefore, the CMoN will depend the type of estimator (e.g., ML) used to obtain parameter estimate. The extrinsic curvature (38) depends on the first and second derivatives of the non-linear function evaluated at the true *x*.

### 4.1. MSE and Sample MSE

We estimate the *x* coordinate using noisy measurements at a discrete set ${\left\{x_{k}\right\}}_{k=1}^{N_{x}}$ of values. Let ${\hat{x}}_{k,m}$ denote the estimate of $x_{k}$ in the *m*th Monte Carlo run. Subsequently, the error ${\tilde{x}}_{k,m}$ in ${\hat{x}}_{k,m}$ is defined by

(77) $${\tilde{x}}_{k,m}:=x_{k}-{\hat{x}}_{k,m},\;k=1,2,\ldots ,N_{x},\;m=1,2,\ldots ,M,$$

where *M* is the number of Monte Carlo runs. The MSE at $x_{k}$ is given by

(78) $${\mathrm{MSE}}_{k}=E\left[{\left({\tilde{x}}_{k,m}\right)}^{2}\right],\;k=1,2,\ldots ,N_{x}.$$

The sample MSE (SMSE) at $x_{k}$ is defined by

(79) $${\mathrm{SMSE}}_{k}:=\frac{1}{M}\sum_{m=1}^{M}{\left({\tilde{x}}_{k,m}\right)}^{2},\;k=1,2,\ldots ,N_{x}.$$

Let $L_{\mathrm{CRLB}}\left(x\right)$ denote the $\log_{10}$ of the CRLB,

(80) $$L_{\mathrm{CRLB}}\left(x\right):=\log_{10}{\mathrm{CRLB}}_{x}.$$

Taking the log of ${\mathrm{CRLB}}_{x}$ in (32) we get

(81) $$L_{\mathrm{CRLB}}\left(x\right)=\log_{10}\left(\frac{\sigma^{2}}{Nn^{2}a^{2}}\right)-2\left(n-1\right)\log_{10}x.$$

### 4.2. MSE and Parameter-Effects Curvature

Let $L_{K}\left(x\right)$ denote the log of the expected value of $K(\hat{x})$ in (57). Then

(82) $$L_{K}\left(x\right):=\log_{10}\left\{E[K\left(\hat{x}\right)]\right\}.$$

In order to compute $L_{K}\left(x\right)$, we first approximate the expectation in (82) by assuming $\sigma_{\hat{x}}\ll x$, which holds for the case investigated in our paper,

(83) $$E\left\{K\left(\hat{x}\right)\right\}=\frac{\left(n-1\right)}{na}E\left(\frac{1}{{\hat{x}}^{n}}\right)\approx \frac{\left(n-1\right)}{na}\frac{1}{{\left[E(\hat{x})\right]}^{n}}\approx \frac{\left(n-1\right)}{na}\frac{1}{x^{n}}.$$

The last step of the above equation follows from an assumption that the estimator is nearly unbiased. Now, taking the logarithm, we have

(84) $$L_{K}\left(x\right)=\log_{10}\left(\frac{n-1}{na}\right)-n\log_{10}x.$$

Now, from Equations (84) and (81), we can see that there is an affine mapping between $L_{\mathrm{CRLB}}\left(x\right)$ and $L_{K}\left(x\right)$. That is,

(85) $$L_{\mathrm{CRLB}}\left(x\right)=\alpha_{1}^{K}L_{K}\left(x\right)+\alpha_{0}^{K},$$

where

(86) $$\begin{array}{c} \begin{array}{cc} \alpha_{1}^{K} & =\frac{2(n-1)}{n},\\ \alpha_{0}^{K} & =\log_{10}\left(\frac{\sigma^{2}}{Nn^{2}a^{2}}\right)-\frac{2(n-1)}{n}\log_{10}\left(\frac{n-1}{na}\right). \end{array} \end{array}$$

We observe that $\alpha_{1}^{K}$ is positive and, hence, $L_{K}\left(x\right)$ and $L_{\mathrm{CRLB}}\left(x\right)$ have the same sign of the non-zero slopes. As a result, $K(\hat{x})$ and CRLB have the same sign of the non-zero slopes.

### 4.3. MSE and Direct Parameter-Effects Curvature

The expression for the direct parameter-effects curvature $\beta_{\delta }\left(\hat{x}\right)$ [29,30] is given by (58).

Similar to the previous section, we define

(87) $$L_{\beta }\left(x\right):=\log_{10}\left(E\{\beta_{\delta }\left(\hat{x}\right)\}\right).$$

Now, taking the expected value of β, we have

(88) $$\begin{array}{c} E\left\{\beta \left(\hat{x}\right)\right\}\approx \frac{\left(n-1\right)}{x}E\left\{|\delta |\right\}=\frac{\left(n-1\right)}{x}E\{|\hat{x}-x\left|\right\}. \end{array}$$

The RHS of (88) can be simplified by assuming that $\hat{x}$ is unbiased and that it achieves the CRLB. Additionally, we approximate this error to be Gaussian and the variance of $\hat{x}$ is given in (29). Then,

(89) $$E\{|\hat{x}-x\left|\right\}=\sqrt{{\mathrm{CRLB}}_{x}}\sqrt{\frac{2}{\pi }}.$$

Substituting (89) into (88) and using (32) for ${\mathrm{CRLB}}_{x}$ we have

(90) $$E\left[\beta \left(\hat{x}\right)\right]\approx \frac{\left(n-1\right)}{na}\sigma \sqrt{\frac{2}{N\pi }}x^{-n}.$$

Thus,

(91) $$L_{\beta }\left(x\right)\approx \log_{10}\left[\frac{\left(n-1\right)\sigma \sqrt{\frac{2}{N\pi }}}{na}\right]-n\log_{10}x.$$

From (91) and (81) we can write the affine mapping

(92) $$L_{\mathrm{CRLB}}\left(x\right)=\alpha_{1}^{\beta }L_{\beta }\left(x\right)+\alpha_{0}^{\beta },$$

where

(93) $$\begin{array}{c} \begin{array}{cc} \alpha_{1}^{\beta } & =\frac{2(n-1)}{n},\\ \alpha_{0}^{\beta } & =\log_{10}\left(\frac{\sigma^{2}}{Nn^{2}a^{2}}\right)-\frac{2n-2}{n}\log_{10}\left[\frac{\left(n-1\right)\sigma \sqrt{2}}{na\sqrt{N\pi }}\right]. \end{array} \end{array}$$

We also observe that $\alpha_{1}^{\beta }$ is positive and, hence, $L_{\beta }\left(x\right)$ and $L_{\mathrm{CRLB}}\left(x\right)$ have the same sign of the non-zero slopes. As a result, $\beta_{\delta }\left(\hat{x}\right)$ and CRLB have the same sign of the non-zero slopes.

### 4.4. Extrinsic Curvature

The expression for extrinsic curvature for our problem is given in (38). Similar to previous sections, we define

(94) $$L_{\kappa }\left(x\right):=\log_{10}\left(\kappa \left(x\right)\right).$$

Taking the log of (94), we have

(95) $$\begin{array}{ccc} L_{\kappa }\left(x\right) & = & \log_{10}\left(\kappa \left(x\right)\right)\\ = & \log_{10}\left[an\left(n-1\right)x^{n-2}\right]-\frac{3}{2}\log_{10}\left[1+{\left(anx^{n-1}\right)}^{2}\right]\\ \approx & \log_{10}\left[an\left(n-1\right)x^{n-2}\right]-\frac{3}{2}\log_{10}\left[{\left(anx^{n-1}\right)}^{2}\right]\\ = & \log_{10}\left[\frac{n-1}{{\left(an\right)}^{2}}\right]-\left(2n-1\right)\log_{10}x. \end{array}$$

Note that the second last expression is a valid approximation for x>2. From (95) and (84) it is easy to establish the affine mapping

(96) $$L_{K}\left(x\right)=\gamma_{1}^{K}L_{\kappa }\left(x\right)+\gamma_{0}^{K},$$

where

(97) $$\begin{array}{c} \begin{array}{cc} \gamma_{1}^{K} & =\frac{n}{2n-1},\\ \gamma_{0}^{K} & =\log_{10}\left(\frac{n-1}{na}\right)-\frac{n}{2n-1}\log_{10}\left[\frac{n-1}{{\left(an\right)}^{2}}\right] \end{array} \end{array}$$

Similarly, from (95) and (91) we can establish the affine relationship

(98) $$L_{\beta }\left(x\right)=\gamma_{1}^{\beta }L_{\kappa }\left(x\right)+\gamma_{0}^{\beta },$$

where

(99) $$\begin{array}{c} \begin{array}{cc} \gamma_{1}^{\beta } & =\frac{n}{2n-1},\\ \gamma_{0}^{\beta } & =\log_{10}\left[\frac{\left(n-1\right)\sigma \sqrt{2}}{na\sqrt{N\pi }}\right]-\frac{n}{2n-1}\log_{10}\left[\frac{n-1}{{\left(an\right)}^{2}}\right]. \end{array} \end{array}$$

Using similar arguments used in previous sections, we infer that the extrinsic curvature and parameter-effects curvature have the same sign of the non-zero slopes. Similarly, the extrinsic curvature and direct parameter-effects curvature have the same non-zero slopes.

### 4.5. Estimation of CMoN and SMSE by Monte-Carlo Simulations

Let $\bar{K}\left({\hat{x}}_{k}\right)$ and ${\bar{\beta }}_{\delta }\left({\hat{x}}_{k}\right)$ denote the sample means of the Bates and Watts and direct parameter-effects curvatures calculated from *M* Monte Carlo runs. Subsequently,

(100) $$\bar{K}\left({\hat{x}}_{k}\right):=\frac{1}{M}\sum_{m=1}^{M}K\left({\hat{x}}_{k,m}\right),\;k=1,2,\ldots ,N_{x},$$

(101) $${\bar{\beta }}_{\delta }\left({\hat{x}}_{k}\right):=\frac{1}{M}\sum_{m=1}^{M}\beta_{\delta }\left({\hat{x}}_{k,m}\right),\;k=1,2,\ldots ,N_{x}.$$

Correspondingly, we define

(102) $$b_{k}:=\log_{10}{\mathrm{SMSE}}_{k},\;k=1,2,\ldots ,N_{x},$$

(103) $$c_{k}:=\log_{10}\bar{K}\left({\hat{x}}_{k}\right),\;k=1,2,\ldots ,N_{x},$$

(104) $$d_{k}:=\log_{10}{\bar{\beta }}_{\delta }\left({\hat{x}}_{k}\right),\;k=1,2,\ldots ,N_{x}.$$

Define

(105) $$b:={\left[\begin{array}{cccc} b_{1} & b_{2} & \ldots & b_{N_{x}} \end{array}\right]}^{\prime },$$

(106) $$c:={\left[\begin{array}{cccc} c_{1} & c_{2} & \ldots & c_{N_{x}} \end{array}\right]}^{\prime },$$

(107) $$d:={\left[\begin{array}{cccc} d_{1} & d_{2} & \ldots & d_{N_{x}} \end{array}\right]}^{\prime }.$$

Suppose that an affine mapping exists between b and c. Subsequently,

(108) $$b_{k}={\hat{\alpha }}_{1}^{K}c_{k}+{\hat{\alpha }}_{0}^{K}+e_{k},\;k=1,2,\ldots ,N_{x},$$

where $e_{k}$ is a random noise. Afterwards, we can write (108) in the matrix-vector form by

(109) $$b=H_{c}\alpha +e,$$

where

(110) $$\alpha :={\left[\begin{array}{cc} {\hat{\alpha }}_{1}^{K} & {\hat{\alpha }}_{0}^{K} \end{array}\right]}^{\prime },$$

(111) $$e:={\left[\begin{array}{cccc} e_{1} & e_{2} & \ldots & e_{N_{x}} \end{array}\right]}^{\prime },$$

(112) $$H_{c}:=\left[\begin{array}{cc} c_{1} & 1\\ c_{2} & 1\\ \ldots & \ldots \\ c_{N_{x}} & 1 \end{array}\right].$$

Given b and $H_{c}$, we can estimate α using the linear least squares (LLS).

We can similarly define the affine mapping between other variable pairs. Altogether, we consider the following four:

1. between b ($\log_{10}\left({\mathrm{SMSE}}_{k}\right)$) and c ($\log_{10}(\bar{K}\left({\hat{x}}_{k}\right)$) for each power of the polynomial function, as in (85),
2. between c ($\log_{10}(\bar{K}\left({\hat{x}}_{k}\right)$) and $\log_{10}\left(\kappa \left(x_{k}\right)\right)$ (94) for each power of the polynomial function, as in (96), and
3. between d ($\log_{10}({\bar{\beta }}_{\delta }\left({\hat{x}}_{k}\right)$) and $\log_{10}\left(\kappa \left(x_{k}\right)\right)$ (94) for each power of the polynomial function, as in (98).

## 5. Numerical Simulation and Results

**Figure 2:**
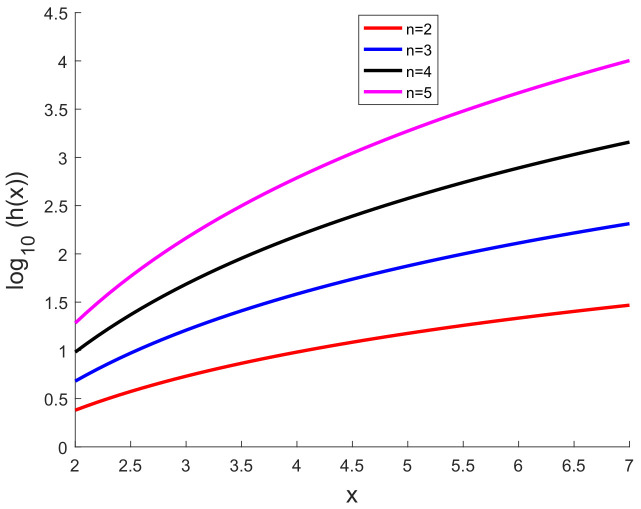
$\log_{10}\left(h\left(x\right)\right)$ versus *x*.

We follow the same simulation scenario as used in our previous work [35]. We use a=0.6 and n=2,3,4,5 and a number of uniformly spaced *x* coordinates with the spacing of 0.1 in the interval [2,7]. The measurement noise standard deviation (σ) is 0.5. The dimension of the measurement vector is 10 or 20. The results are based on 1000 Monte Carlo runs. Figure 2 shows $\log_{10}\left(h\left(x\right)\right)$ versus *x*.

To assess the accuracy of the MLE, we compute the sample bias, sample MSE, ANEES [42], and CRLB [2,41,51]. Let $x_{k,i}=x_{k}$, ${\hat{x}}_{k,i}$, and $\sigma_{k,i}^{2}$ denote the true parameter, ML estimate, and associated variance, respectively, at the *k*th point in the *i*th Monte Carlo run. The sample bias in the estimate at the *k*th point is defined by [9]

(113) $${\hat{b}}_{k}:=\frac{1}{M}\sum_{i=1}^{M}\left(x_{k,i}-{\hat{x}}_{k,i}\right),$$

where *M* is the number of Monte Carlo runs. The sample root MSE (RMSE) [9] and ANEES [2,9,42] at the *k*th point are defined, respectively, by

(114) $${\mathrm{RMSE}}_{k}:={\left[\frac{1}{M}\sum_{i=1}^{M}{\left(x_{k,i}-{\hat{x}}_{k,i}\right)}^{2})\right]}^{1/2},$$

(115) $${\mathrm{ANEES}}_{k}:=\frac{1}{M}\sum_{i=1}^{M}{\left(x_{k,i}-{\hat{x}}_{k,i}\right)}^{2}/\sigma_{k,i}^{2}.$$

**Figure 3:**
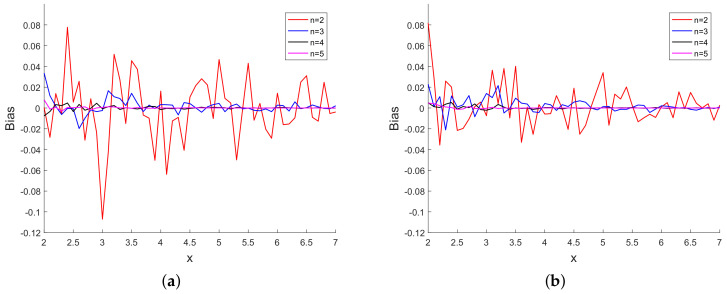
(**a**) Sample bias vs. *x* using 10 scalar measurements and (**b**) sample bias vs. *x* using 20 scalar measurements.

**Figure 4:**
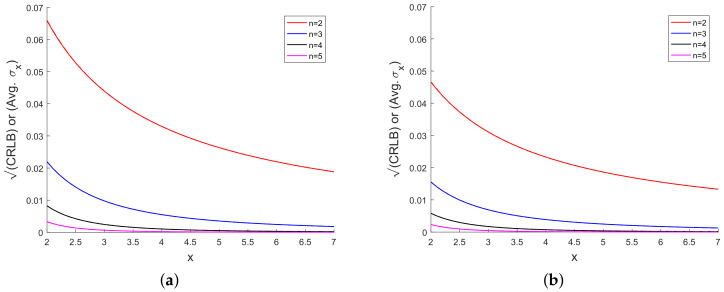
(**a**) $\sqrt{\mathrm{CRLB}}$ or (Avg. $\sigma_{x}$) vs. *x* using 10 scalar measurements and (**b**) $\sqrt{\mathrm{CRLB}}$ or (Avg. $\sigma_{x}$) vs. *x* using 20 scalar measurements.

Figure 3 presents the sample bias for different powers of *x*. We observe from Figure 3 that the bias is small when compared with the true value of *x* and the bias decreases with increase in the power of x. In Figure 4, we have plotted the $\sqrt{\mathrm{CRLB}}$ and the average of $\sigma_{x}$ over Monte Carlo runs. Figure 4 shows that, for each power of *x*, the $\sqrt{\mathrm{CRLB}}$ and the average of $\sigma_{x}$ are on top of each other and it is not possible to distinguish them in the figure.

**Figure 5:**
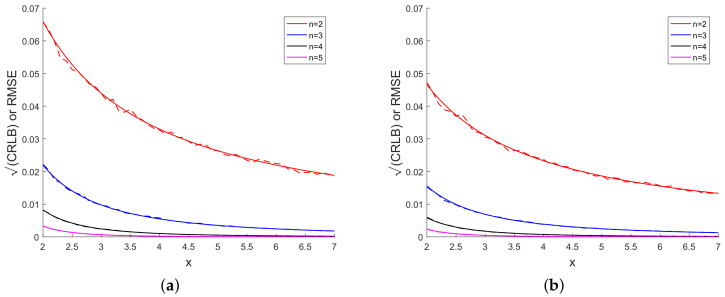
(**a**) $\sqrt{\mathrm{CRLB}}$ or RMSE vs. *x* using 10 scalar measurements and (**b**) $\sqrt{\mathrm{CRLB}}$ or RMSE vs. *x* using 20 scalar measurements.

Figure 5 presents $\sqrt{\mathrm{CRLB}}$ and RMSE for each power of *x*. Solid and dashed lines in Figure 5 represent the $\sqrt{\mathrm{CRLB}}$ and RMSE, respectively, for each power of *x*. We see from Figure 5 that corresponding values of $\sqrt{\mathrm{CRLB}}$ and RMSE are close to each other for each power of *x*. In Figure 3, Figure 4 and Figure 5, the bias, $\sqrt{\mathrm{CRLB}}$, $\sigma_{x}$ and RMSE for 20 measurements are smaller than corresponding values for 10 measurements.

**Figure 6:**
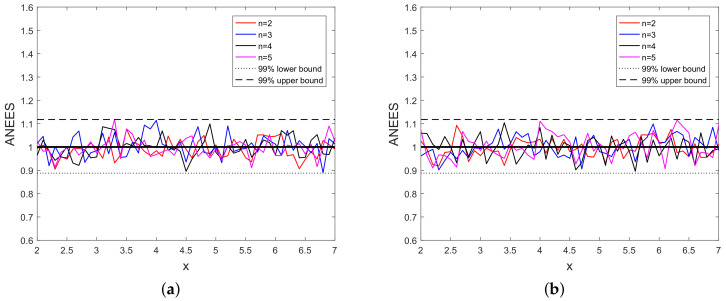
(**a**) ANEES vs. *x* using 10 scalar measurements and (**b**) ANEES vs. *x* using 20 scalar measurements.

We present the ANEES [42] in Figure 6 for different powers of *x* with 99% confidence bounds. We see from Figure 6 hat the ANEES lies within the 99% confidence bounds. This shows that the variance $\sigma_{x}^{2}$ calculated using the MLE is consistent with the estimation error.

**Figure 7:**
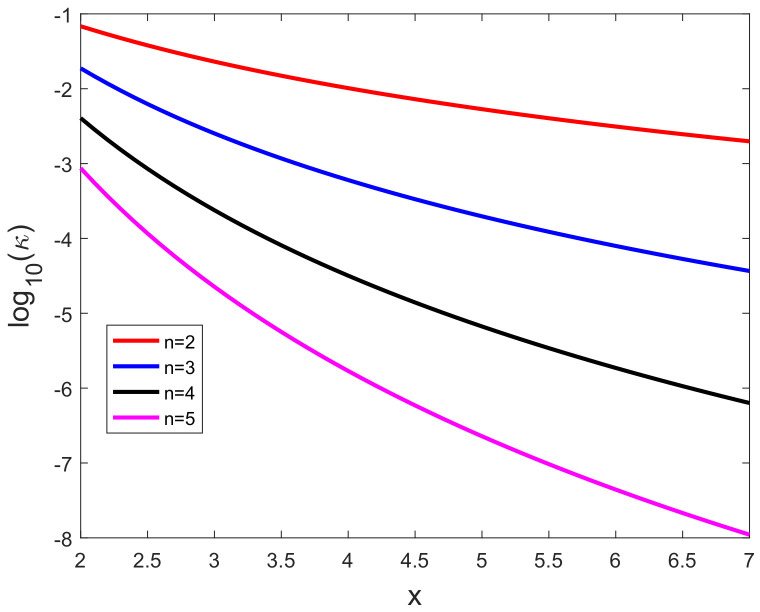
Logarithm of the extrinsic curvature $\log_{10}$(*k*(*x*)) versus *x*.

Figure 7 presents the logarithm of the extrinsic curvature $\log_{10}\left(\kappa \left(x\right)\right)$ versus *x*. The extrinsic curvature is completely determined by the first and second derivatives of the non-linear function *h* and it is evaluated while using the true *x*.

**Figure 8:**
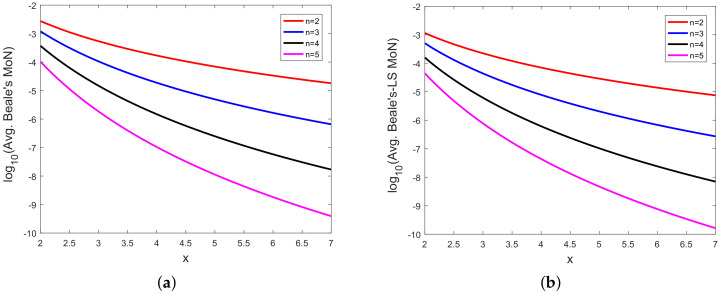
(**a**) Logarithm of Beale’s MoN ($\log_{10}$(Avg. Beale’s MoN)) vs. *x* and (**b**) logarithm of Beale’s MoN using LS ($\log_{10}$(Avg. Beale’s-LS MoN)) vs. *x* with 10 scalar measurements.

**Figure 9:**
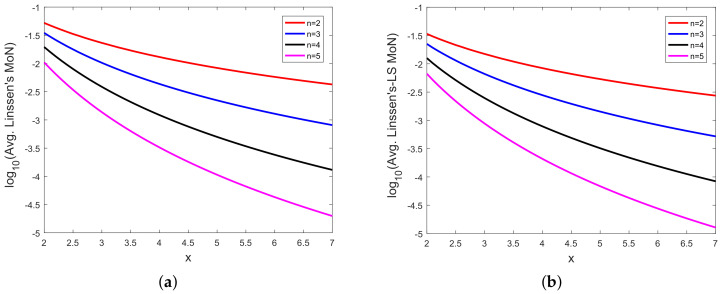
(**a**) Logarithm of Linssen’s MoN ($\log_{10}$(Avg. Linssen’s MoN)) vs. *x* and (**b**) logarithm of Linssen’s MoN using LS ($\log_{10}$(Avg. Linssen’s-LS MoN)) vs. *x* with 10 scalar measurements.

**Figure 10:**
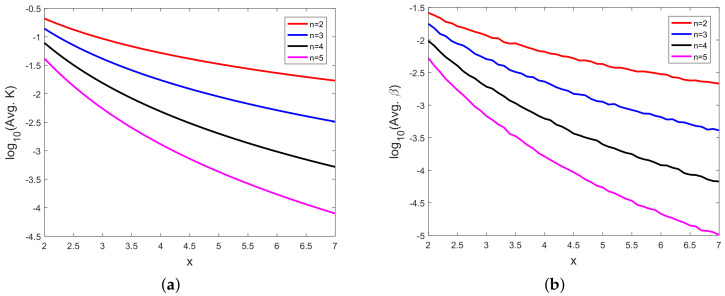
(**a**) Logarithm of Bates and Watts parameter-effects curvature ($\log_{10}$(Avg. *K*)) vs. *x* and (**b**) logarithm of direct parameter-effect curvature ($\log_{10}$(Avg. *β*)) vs. *x* using 10 scalar measurements.

**Figure 11:**
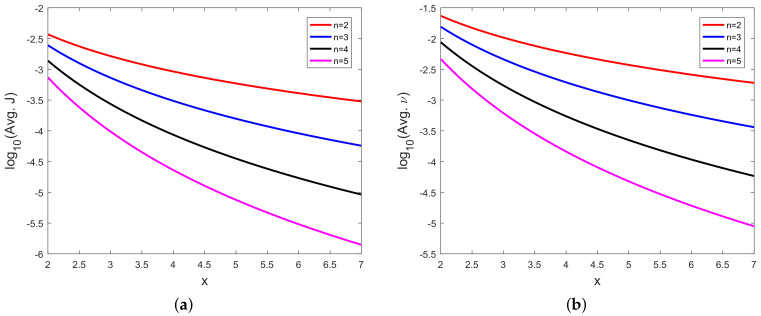
(**a**) Logarithm of Li’s un-normalized MoN ($\log_{10}$(Avg. *J*)) vs. *x* and (**b**) logarithm of Li’s normalized MoN ($\log_{10}$(Avg. *J*)) vs. *x* with 10 scalar measurements.

**Figure 12:**
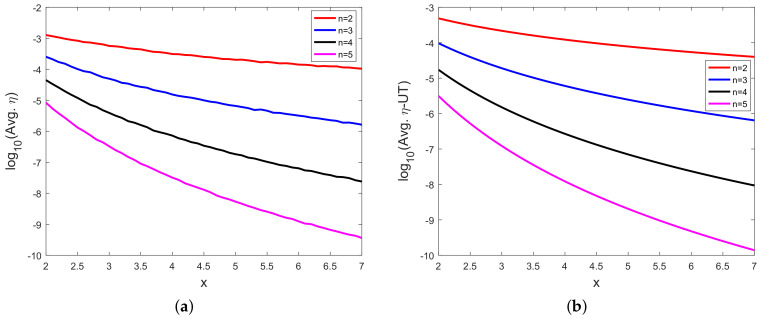
(**a**) Logarithm of MoN of Straka et al. ($\log_{10}$(Avg. η)) vs. *x* and (**b**) logarithm of MoN of Straka et al. with UT ($\log_{10}$(Avg. η-UT)) vs. *x* using 10 scalar measurements.

**Figure 13:**
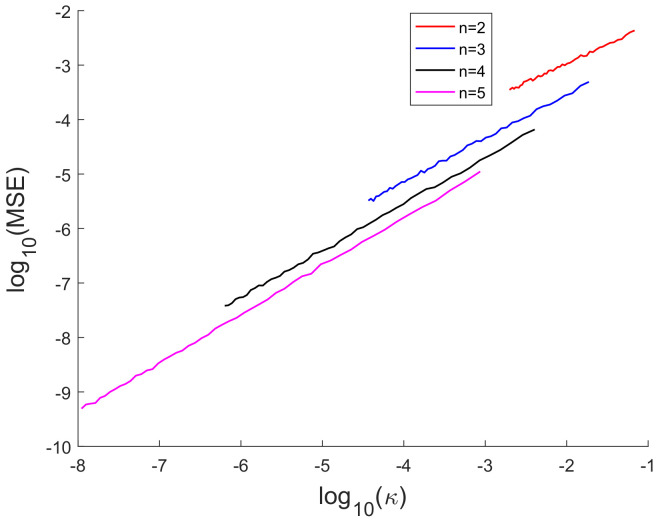
$\log_{10}$(MSE) vs. logarithm of extrinsic curvature ($\log_{10}$ (κ)) using 10 scalar measurements.

**Figure 14:**
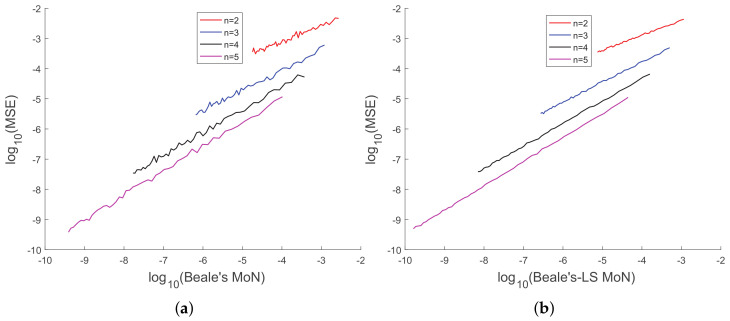
(**a**) $\log_{10}$(MSE) vs. $\log_{10}$(Avg. Beale’s MoN) and (**b**) $\log_{10}$(MSE) vs. $\log_{10}$ (Avg. Beale’s MoN using LS) using 10 scalar measurements.

**Figure 15:**
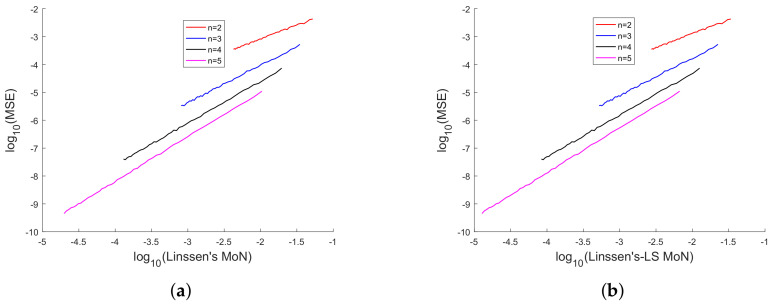
(**a**) $\log_{10}$(MSE) vs. $\log_{10}$(Linssen’s MoN) and (**b**) $\log_{10}$(MSE) vs. $\log_{10}$ (Linssen’s-LS) using 10 scalar measurements.

**Figure 16:**
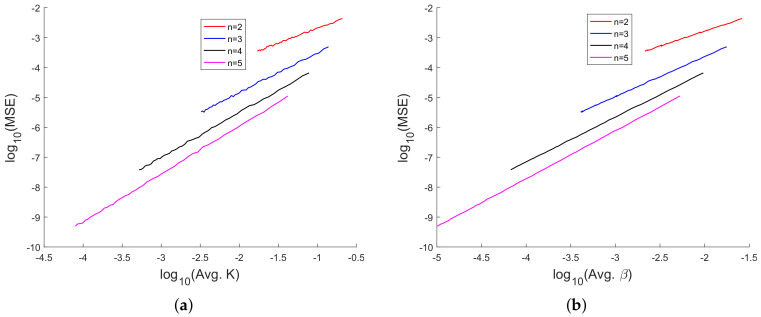
$\log_{10}$(MSE) vs. logarithm of parameter-effects curvatures. (**a**) $\log_{10}$(MSE) vs. $\log_{10}$(Avg. *K*) and (**b**) $\log_{10}$(MSE) vs. $\log_{10}$ (Avg. β) using 10 scalar measurements.

**Figure 17:**
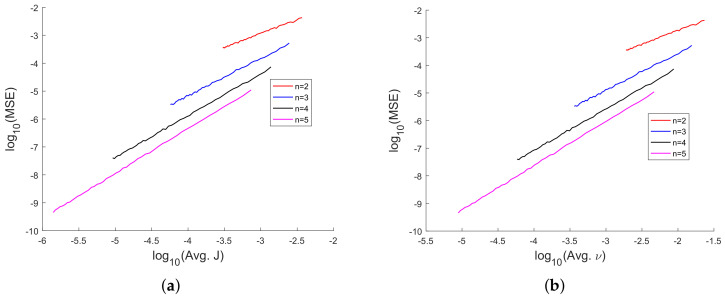
$\log_{10}$(MSE) vs. logarithm of Li’s MoN. (**a**) $\log_{10}$(MSE) vs. $\log_{10}$(Avg. *J*) and (**b**) $\log_{10}$(MSE) vs. $\log_{10}$ (Avg. ν) using 10 scalar measurements.

**Figure 18:**
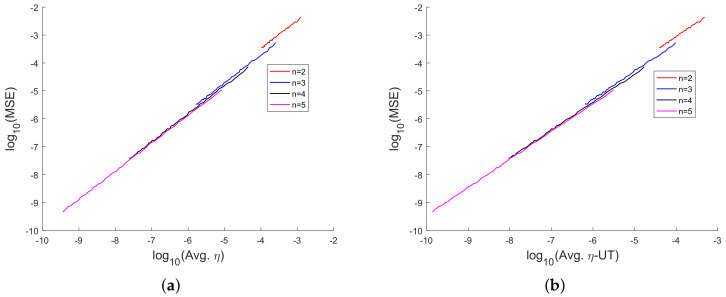
$\log_{10}$(MSE) vs. logarithm of MoN of Straka et al. (**a**) $\log_{10}$(MSE) vs. $\log_{10}$(Avg. η) and (**b**) $\log_{10}$(MSE) vs. $\log_{10}$ (Avg. η-UT) using 10 scalar measurements.

In Figure 8, Figure 9, Figure 10, Figure 11, Figure 12, Figure 13, Figure 14, Figure 15, Figure 16, Figure 17 and Figure 18, we present results using 10 scalar measurements. We have also generated results using 20 scalar measurements. In order to limit the number of figures, we have not presented figures with 20 scalar measurements. The CRLB, variance of estimation error, all MoNs, and MSE follow the same trend. However, the corresponding values compared with 20 measurements are reduced due to improved estimation accuracy.

In [35], we had shown analytically, and through Monte Carlo simulation, that affine mappings exist among $\log_{10}$(MSE), $\log_{10}$ (κ), $\log_{10}$(Avg. *K*), and $\log_{10}$ (Avg. β). In Figure 13, Figure 14, Figure 15, Figure 16, Figure 17 and Figure 18, we have plotted the $\log_{10}$(MSE) versus $\log_{10}$ of various MoNs using 10 scalar measurements. These figures show that the $\log_{10}$(MSE) varies with $\log_{10}$ (MoN) according to an affine mapping with a positive slope. This implies that the MSE increases as an MoN increases. We obtain similar results for the case of 20 scalar measurements.

The above results demonstrate that, for the polynomial nonlinearity problem analyzed, any of the seven MoNs analyzed is suitable metrics to quantify the MSE, which represents the complexity of a parameter estimation problem. Further research is needed to study the applicability of these MoNs in real-world non-linear filtering problems.

## 6. Conclusions

We considered a polynomial curve in 2D and derived analytic expressions for the ML estimate and associated variance of the independent variable *x* using a vector measurement. The ML estimate is used to evaluate the Jacobian and Hessian of the measurement function appearing in the computation of Bates and Watts and direct parameter-effects curvatures, Beale’s MoN, and Linssen’s MoN. Our numerical results show that the variance of the estimated parameter and the Cramér-Rao lower bound (CRLB) are nearly the same for different powers of *x*. The average normalized estimation error squared (ANEES) lies within the 99% confidence interval, which indicates that the ML based variance is consistent with the estimation error.

We used seven MoNs, including the extrinsic curvature using differential geometry, Beale’s MoN (and its least squares variant), Linssen’s MoN (and its least squares variant), Bates and Watts parameter-effects curvature, direct parameter-effects curvature, Li’s MoN, and the MoN of Straka, Duník, and S̆imandl. If a MoN has a high value, then the nonlinearity is high. All of the MoNs show the same type of variation with *x* and the power of of the polynomial. Secondly, as the logarithm of a MoN increases, the logarithm of the MSE also increases linearly for each MoN. This implies that, as a MoN increases, and then the MSE increases. These results are quite surprising, given the fact that these MoNs are derived based on completely different theoretical considerations. The second feature of our analysis is useful in establishing that a MoN in our study can be considered as a candidate metric for quantifying the MSE that represents the complexity of a parameter estimation problem. Our future work will study other practical parameter estimation and non-linear filtering problems.

## Figures and Tables

**Table 1:** Features of Various MoNs

MoN	Parameters Used	Local/Global	Need?			Basic Idea	Random?
			Measurements	Jacobian/Hessian	Covariances		
Extrinsic Curvature	True	Local	No	Jacobian and Hessian at true value	No	Differential Geometry	Non-random
Beale’s MoN	True & estimated	Local	Yes	Jacobian	No	Scaled sum square distance	Random
Linssen’s MoN	True & estimated	Local	Yes	Jacobian	No	Root scaled sum square distance	Random
Parameter-effects Curvature	True & estimated	Local	Yes	Jacobian and Hessian at estimated value	No	Differential Geometry	Random
Li’s MoN	True	Global	Yes	No	Yes	min. mean square distance	Random
MoN by Straka et al.	True & estimated	Local	Yes	No	No	WLS cost function	Random

**Table 2:** MoNs for the bearing-only, GMTI, and video filtering problems.

Curvature Type	Bearing-Only	GMTI	Video
Parameter-effects	(300–1200) × 10${}^{-4}$	(0.8–1.2) × 10${}^{-4}$	0.245 × 10${}^{-4}$
Intrinsic	(69–149) × 10${}^{-4}$	0.2 × 10${}^{-4}$	0.066 × 10${}^{-4}$
Total	(369–1349) × 10${}^{-4}$	(1.0–1.4) × 10${}^{-4}$	0.312 × 10${}^{-4}$
